# Relative emissions intensity of dairy production systems: employing different functional units in life-cycle assessment

**DOI:** 10.1017/S1751731117000052

**Published:** 2017-02-10

**Authors:** S. A. Ross, C. F. E. Topp, R. A. Ennos, M. G. G. Chagunda

**Affiliations:** 1 Future Farming Systems Group, SRUC, EH9 3JG Edinburgh, UK; 2 Crop and Soil Systems Systems Group, SRUC, EH9 3JG Edinburgh, UK; 3 School of Biological Sciences, University of Edinburgh, EH9 3FF Edinburgh, UK

**Keywords:** life-cycle assessment, functional unit, dairy cow, greenhouse gas

## Abstract

This study aimed to assess the merit and suitability of individual functional units (FU) in expressing greenhouse gas emissions intensity in different dairy production systems. An FU provides a clearly defined and measurable reference to which input and output data are normalised. This enables the results from life-cycle assessment (LCA) of different systems to be treated as functionally equivalent. Although the methodological framework of LCA has been standardised, selection of an appropriate FU remains ultimately at the discretion of the individual study. The aim of the present analysis was to examine the effect of different FU on the emissions intensities of different dairy production systems. Analysis was based on 7 years of data (2004 to 2010) from four Holstein-Friesian dairy systems at Scotland’s Rural College’s long-term genetic and management systems project, the Langhill herd. Implementation of LCA accounted for the environmental impacts of the whole-farm systems and their production of milk from ‘cradle to farm gate’. Emissions intensity was determined as kilograms of carbon dioxide equivalents referenced to six FU: UK livestock units, energy-corrected milk yield, total combined milk solids yield, on-farm land used for production, total combined on- and off-farm land used for production, and the proposed new FU–energy-corrected milk yield per hectare of total land used. Energy-corrected milk was the FU most effective for reflecting differences between the systems. Functional unit that incorporated a land-related aspect did not find difference between systems which were managed under the same forage regime, despite their comprising different genetic lines. Employing on-farm land as the FU favoured grazing systems. The proposed dual FU combining both productivity and land use did not differentiate between emissions intensity of systems as effectively as the productivity-based units. However, this dual unit displayed potential to quantify in a simple way the positive or negative outcome of trade-offs between land and production efficiencies, in which improvement in emissions intensity using one FU may be accompanied by deterioration using another FU. The perceived environmental efficiencies of different dairy production systems in terms of their emissions intensities were susceptible to change based upon the FU employed, and hence the FU used in any study needs to be taken into account in the interpretation of results.

## Implications

Dairy production systems are key contributors of greenhouse gas (GHG) emissions. Emissions intensity is estimated by life-cycle assessment (LCA) and is influenced by different feeding and management systems and dairy cows. The perceived environmental efficiency of different dairy systems can change depending on the functional unit (FU) to which emissions are referenced. Results from studies comparing the emissions intensity of different dairy production systems should be considered in context of the FU used, in order to appraise them in an informed manner.

## Introduction

Life-cycle assessment has become a leading tool employed in agriculture for environmental impact and GHG emissions accounting at the whole-systems level. Favoured for its flexibility, LCA enables an account to be made of all system inputs, processes and outputs within a specified boundary. In order to improve transparency and consistency amongst studies, the international standard ISO 14040 was established (ISO, 2006), stipulating requirements and recommendations for the LCA decision-making process. Further, frameworks attempting to institute consistency in LCA at national and industry-specific levels have been developed, such as PAS 2050 in the United Kingdom (BSI, 2011). By convention, LCA results must be referenced to an FU, providing a clearly defined and measurable reference to which input and output data are normalised (ISO, 2006). For the purposes of GHG measurement, the FU can either be a single item of product or a generally accepted sales quantity (BSI, 2011). The selection of an appropriate FU is crucial when assessing and interpreting environmental impacts, in which an impact category such as GHG emissions may be referenced to several different FUs (Haas *et al*., 2001).

In LCA of dairy production systems, the FU has most frequently been a unit of milk (Yan *et al*., 2011; Baldini *et al*., 2016), the principal unit of production in dairy. Milk yields have commonly been corrected to standardised levels of milk fat and protein content, as milk composition commonly determines the value of raw milk to the processor. This enables better comparison between farms with different breeds or different feeding regimes (IDF, 2010). Other production-based FUs have been employed, including the combined mass of milk fat and protein as milk solids (MS), or simply as uncorrected raw milk yields (Baldini *et al*., 2016). When the LCA boundary included post-farm processing of dairy products, results have been referenced to a unit of consumer product such as processed packaged milk (Hospido *et al*., 2003). Expressing emissions intensity per unit of productivity is essentially a ratio of undesirable *v*. desirable system outputs. Land use is also commonly used and satisfies a more conventional definition of efficiency, being a measure of system output *v*. system input. As an FU, land use can include the productive on-farm land required for grazing and forage production or the entire farm area. Recently, an area-based FU has also incorporated off-farm land required for production of purchased feeds (O’Brien *et al*., 2012). The livestock themselves, expressed as the total number of livestock units (LSU) on the farm, has also been used as an FU (Haas *et al*., 2001). Although concerted efforts have been made to establish consistency in the application of LCA to dairy production systems, selection of an FU ultimately remains at the discretion of individual investigators.

Studies directly examining the effect of varying the FU on the results of LCA of different dairy production systems are sparse. Several studies have employed multiple FUs, therefore indirectly providing some assessment, although not necessarily as the primary aim (e.g. Haas *et al*., 2001; O’Brien *et al*., 2012). Results of these studies indicate that the perceived relative environmental efficiency of dairy production systems can change on the basis of FU employed. The objective of our study was to examine the effect of employing different FU on the estimated environmental efficiency of different dairy production systems and hence the ability to classify different production systems on the basis of GHG emissions intensity.

## Material and methods

### Dairy production systems and life-cycle assessment

The study was based on data from Scotland’s Rural College’s (SRUC) long-term genetic and management systems project, the Langhill herd, located at the SRUC’s Dairy Research Centre, Crichton Royal Farm, Dumfries, Scotland. Data used were collected from January 2004 to December 2010 from four distinct systems within a conventional farm. Holstein-Friesian cows were maintained in two feeding groups: high forage (HF) and low forage (LF). The HF systems aimed to provide 75% by dry matter of the herd’s total mixed ration (TMR) diet from home-grown crops (i.e. ryegrass silage, whole-crop maize, wheat alkalage) and 25% from purchased concentrated feeds (e.g. distillers grains, rapeseed meal). Cows in the HF systems were also put outside to graze pasture, when available. In contrast, cows in the LF systems were fully housed and fed a diet of ~45% of the same home-grown forages and 55% purchased concentrated feeds. Within each forage system, animals comprised two contrasting genetic lines. Control (C) animals were bred to be of average UK genetic potential for milk fat and protein production, whereas Select (S) animals represented the top 5% of UK genetic potential. Maintaining the specific characteristics of these groups in a long-term genotype×feeding regime project resulted in four divergent dairy production systems: HFC, HFS, LFC and LFS. Cows were milked three times daily and received equal health and fertility treatments, and herd size was maintained at ~50 cows per system. Control and S cows were managed together, and groups were kept in the same building when housed. All rations were formulated using the same preserved forages, and all young stock were managed together (Chagunda *et al*., 2009).

Annual GHG emissions were estimated using LCA, covering all stages of dairy production from the extraction or acquisition of raw materials up to the point at which the product milk left the farm. In brief, high-resolution data were collected for on-farm processes, including herd dynamics, electricity and fuel use, crop production, fertiliser application, as well as animals’ diet formulation, feed intake and productivity. Impacts were assessed by applying Intergovernmental Panel on Climate Change (IPCC) Tier 2 methodology (IPCC, 2006). Intergovernmental Panel on Climate Change coefficients were used to estimate direct emissions, volatilisation and leaching associated with application and storage of fertiliser and manure, and from crop residues (IPCC, 2006). United Kingdom emission factors were used for production and transport of fertilisers, concentrated feeds and animal bedding (Carbon Trust, 2010), and for electricity and fuel use (DEFRA, 2011). System-specific Tier 3 emissions factors were estimated for enteric methane, manure methane and excreted nitrogen (Ross *et al*., 2014). Life-cycle emissions were allocated between two system outputs – the products milk and meat – using mass allocation. Emissions intensity was defined as the estimated global warming potential (GWP) of each system in kilogram of carbon dioxide equivalents (kg CO_2_e) per FU. Additional in-depth details of the LCA performed are described in Ross *et al*. (2014).

### Functional units

The estimated annual GWP of dairy production systems was referenced to five FU employed in previous LCA studies of dairy production systems and to one further FU which incorporated a measure of both the productivity and land use of the systems (Table 1).

**Table 1:** Functional units employed in the life-cycle assessment

Functional unit	Abbreviation	Unit
United Kingdom livestock unit	LSU	n
Energy-corrected milk yield	ECM	kg
Total combined milk solids yield	MS	kg
On-farm land used for production	Land_farm_	ha
Combined on- and off-farm land used for production	Land_total_	ha
Energy-corrected milk per unit of total land used	ECM/Land_total_	t/ha

#### Livestock units

At system level, the LCA included not only milking cows but also all other age classes of livestock. Employing LSU enabled the entire herd to be included in the FU, applying a conversion factor for the relative ages and persistence of young and replacement animals. These were defined for calves aged 0 to 12 months, heifers 12 to 24 months and heifers over 24 months as 0.34, 0.65 and 0.80, respectively (DEFRA, 2010). Milking cows in different systems were corrected for average BW and milk yield (ADAS, 1983), with values of 1.48, 1.64, 1.26 and 1.37 for LFC, LFS, HFC and HFS, respectively. All male calves were sold after weaning, and there were no bulls on the farm. Total annual populations of livestock were multiplied by their respective coefficients and summed to give an average population in LSU for each system.

#### Energy-corrected milk

Milk yields amongst the production systems varied in energy content, which is defined by fat and protein contents (Table 2), potentially leading to differences in calculations if only the mass or volume of milk was considered. We used the equation of Sjaunja *et al*. (1990), commonly employed in LCA, to calculate energy-corrected milk (ECM) with 35.0 g/kg milk fat and 32.0 g/kg protein as follows: ECM (kg)=0.25M+12.2F+7.7P, where M is the annual milk yield (kg), F the milk fat content (kg), P the protein content (kg).

**Table 2:** Average milk composition and yield of Langhill dairy production systems (mean±SD)

	Production system			
Characteristics	LFC	LFS	HFC	HFS
Milk fat (g/kg)	35.4±0.18	37.8±0.18	38.2±0.18	40.0±0.19
Milk protein (g/kg)	31.5±0.09	33.7±0.09	32.1±0.09	33.5±0.01
Annual milk yield (kg/cow)	9246±800	10 753±583	7281±533	8189±656

LFC=low forage control; LFS=low forage select; HFC=high forage control; LFS=high forage select.

#### Milk solids

Data on milk yield were recorded for individual cows after each milking session. Milk fat and protein contents were recorded from samples collected from each cow, three times daily on 1 day each week. An estimate was thus made for the total mass of milk fat and protein yielded by each cow per calendar year. Individual cow data were combined to give the total annual output of MS for each system.

#### On-farm land use

On-farm land use to provide forage crops (ryegrass silage, whole-crop wheat, maize) to each system was estimated on the basis of forage requirements of the system. Quantities of ensiled forages used were weighed during formulation of the daily TMR, and daily feed intake of cows was recorded using automated Hoko feeding gates (Insentec BV, Marknesse, the Netherlands). Forage crop requirements of each system were then related to harvested forage yields and estimated annual forage land requirements. Dry matter losses were considered during harvesting, ensiling and unloading of forages (Bastiman and Altman, 1985; MacDonald *et al*., 1991). Land required by HF systems for pasture was similarly estimated based upon the predicted grazing dry matter intake (DMI) of cows and the available herbage per hectare (Table 3). Applying the estimates from Bell *et al*. (2010), DMI of HF cows grazing pasture was 19.2 kg/day for C cows and 20.8 kg/day for S cows.

**Table 3:** Crop yields and on-farm land use by Langhill systems (mean±SD)

	Grass silage	Maize silage	Wheat	Pasture
Crop yield (t DM/ha)	10.3±1.5	11.9±2.1	11.6±2.3	10.3±1.5
Land use by system (ha)				
LFC	18.1±4.0	4.0±0.8	3.2±0.7	
LFS	18.0±4.2	4.2±0.7	3.4±0.9	
HFC	18.2±4.1	4.1±0.9	3.3±0.7	11.9±2.8
HFS	18.2±4.2	4.2±0.8	3.4±0.8	12.2±3.0

DM=dry matter; LFC=low forage control; LFS=low forage select; HFC=high forage control; LFS=high forage select.

#### Off-farm land use

Off-farm land associated with the external production of purchased feed components and bedding was estimated using the method of Bell *et al*. (2011) (Table 4). First, total annual purchased feed required was estimated from recorded Hoko data and from TMR formulations, breaking the purchased fraction of the diets down into component ingredients. Second, land use values for domestically produced purchased feed components (wheat, rapeseed, barley) were estimated using national data on crop yields (Craig and Logan, 2012; Scottish Government, 2012). Third, land use for the internationally imported soyabean meal was sourced from the LCA food database (Nielsen *et al*., 2007). Finally, allocations between co-products of purchased feed components were made using mass-based factors from Cederberg and Mattsson (2000).

**Table 4:** Breakdown of components in purchased feed blends and estimated land use

	Barley	Wheat	Sugar beet pulp	Soyabean meal	Rapeseed meal	Complete blend
Whole crop yield (t DM/ha)	5.9	7.0	10.0	3.0	3.9	
Allocation (%)^1^	100	100	22	80	60	
Percentage in blend						
Low forage		47	28	25		
High forage	33.3	33.3			33.3	
Land use (m^2^/kg)						
Low forage		0.64	0.06	0.75		1.44
High forage	0.43	0.43			0.70	1.55

DM=dry matter.

1 Percentage of environmental impact attributed to production of each component of feed blend by mass allocation (Cederberg and Mattsson, 2000).

#### Dual functional unit

In this study we introduce a new FU including both the productivity and land use of systems. The estimated annual GWP of the dairy production system was divided by the total annual ECM yield, and this quotient further divided by the total on- and off-farm land use of the system for each year of the study. This dual FU thus incorporated the ratio of undesirable output (GHG) to desirable output (milk) per unit input (area of land) and therefore adhered to a more standardised output/input measure of efficiency.

### Statistical analysis

Two statistical procedures were employed in the analysis. The effect of employing different FU upon the estimated environmental efficiency of dairy production systems was assessed using ANOVA employing the following GLM, *y*_*ij*_=*µ*+*S*_*i*_+*Y*_*j*_+*ε*_*ij*_ where *y*_*ij*_ was the total GWP of the dairy production system per FU (LSU, ECM, MS, Land_farm_, Land_total_, ECM/Land_total_), *µ* the overall mean, *S*_*i*_ the fixed effect of dairy production system (LFC, LFS, HFC, HFS), *Y*_*j*_ the random effect of calendar year (2004–10) and *ε*_*ij*_ was the random error term. Fisher’s tests were used to assess the level of significance of contributing effects, and differences between dairy production systems were determined by conducting pairwise comparisons using the Tukey’s method.

The ability of different FU to classify different production systems’ relative environmental efficiency was assessed using rank analysis. Using year as a repeated measure, systems were assigned a rank value from 1 to 4 in order of their relative emissions intensity, with rank 1 having the lowest GWP per FU and thus most efficient system. Rank analysis was performed using the Kruskal–Wallis’s test, a non-parametric equivalent to ANOVA. Significant differences between any two systems were assessed using the Mann–Whitney–Wilcoxon’s rank-sum test. The rank analysis was repeated when referencing the GWP of systems to each of the six FU employed in this study. All statistical analyses were conducted using Minitab 16.

## Results

### Emissions intensity

The effect of the dairy production system on the overall GWP per FU was significantly different (*P*<0.001) for each of the six FU. Energy-corrected milk was the only FU for which the GWPs of all four systems were significantly different from each other (*P*<0.001) (Table 5). Low forage select animals had the lowest GWP per kilogram ECM, followed by LFC, HFS and HFC. Using MS as the FU, LFS again had the lowest GWP and HFC the highest. There was no significant difference between LFC and HFS per kilogram of MS. Using LSU as the FU, LFC was the most efficient, although not significantly different from LFS. Livestock units was the only FU which did not find a significant difference between LFS and HFC. Using Land_farm_ as the FU, GWP per hectare of both HF systems was lower than those of both LF systems. Conversely, including off-farm land use (Land_total_), both LF systems had lower GWP per hectare than both HF systems. Using the dual FU, which incorporated both productivity and land use, both LF systems were more efficient than both HF systems. However, none of the three FU which incorporated land use found a significant difference between HFC and HFS or between LFC and LFS. Overall, LFS was the most efficient system for four of the six FU (ECM, MS, Land_total_, ECM/Land_total_).

**Table 5:** Emissions intensity of Langhill dairy production systems expressed as kilograms of carbon dioxide equivalents (kg CO_2_e) per functional unit

	Production system					
Functional unit	LFC	LFS	HFC	HFS	SEM	*P-*value
LSU (*n*)	4126^a^	4398^ab^	4535^bc^	4807^c^	126.3	***
ECM (kg)	0.92^a^	0.83^b^	1.10^c^	1.00^d^	0.016	***
MS (kg)	12.9^a^	11.4^b^	15.2^c^	13.7^a^	0.23	***
Land_farm_ (ha)	16 006^a^	15 971^a^	11 506^b^	11 704^b^	252.5	***
Land_total_ (ha)	6287^a^	6304^a^	8041^b^	7467^b^	236.2	***
ECM/Land_total_ (t/ha)	14.9^a^	13.4^a^	21.4^b^	20.5^b^	0.82	***

LFC=low forage control; LFS=low forage select; HFC=high forage control; LFS=high forage select; LSU=livestock units; ECM=total energy-corrected milk yield; MS=total milk solids; Land_farm_=on-farm land use; Land_total_=total land use; ECM/Land_total_=milk yield per unit total land use. ^a,b,c,d^Values within a row with different superscripts differ significantly at ***P<0.001.

### Rank analysis

For each of the six FU, median rankings of environmental efficiency of dairy production systems were significantly different (*P*<0.05) (Table 6). The median rankings broadly reflected the relative order of system efficiency observed from ANOVA results, with two differences. Using Land_total_ as the FU, HFS was the lowest ranked system and significantly different from the 3^rd^ ranked HFC. Furthermore, the two most efficient systems when using the dual FU, LFS and LFC, were significantly different from each other according to the Mann–Whitney–Wilcoxon’s rank-sum test, with LFS having the lower GWP. However, when using LSU or Land_farm_ as the FU, LFS was ranked 4^th^.

**Table 6:** Median rankings denoting the relative emissions intensities of Langhill dairy production systems

	Production system				
Functional unit	LFC	LFS	HFC	HFS	*P-*value
LSU (*n*)	1^a^	2^b^	3^b^	4^c^	*
ECM (kg)	2^a^	1^b^	4^c^	3^d^	*
MS (kg)	2^a^	1^b^	4^c^	3^a^	*
Land_farm_ (ha)	3^a^	4^a^	1^b^	2^b^	*
Land_total_ (ha)	1^a^	2^a^	3^b^	4^c^	*
ECM/Land_total_ (t/ha)	2^a^	1^b^	4^c^	3^c^	*

LFC=low forage control; LFS=low forage select; HFC=high forage control; LFS=high forage select; LSU=livestock units; ECM=energy-corrected milk yield; MS=total milk solids; Land_farm_=on-farm land use; Land_total_=total land use; ECM/Land_total_=milk yield per unit total land use. ^a,b,c,d^Values within a row with different superscripts differ significantly at *P<0.05.

## Discussion

Livestock units are commonly used in the dairy industry to compare stocking densities and nutritional requirements of animals; however, LSU have been infrequently used when interpreting outputs of LCA studies. When using LSU as the FU in this study, the LF systems had the lowest emissions intensity. When Haas *et al*. (2001) referenced emissions to LSU (defined as 500 kg of cow BW), extensive grazing-based systems had lower emissions than intensive systems. This trend was not reflected in our results. Given the higher gross emissions of the LF regime (Ross *et al*., 2014) and that systems had similar numbers of milking cows, the difference observed was likely due to greater animal performance under LF. The UK LSU is based on a standard of 48 000 MJ of metabolisable energy, defined as ‘the feed energy allowance of a 625 kg Friesian cow and the production of a 40 kg calf, and 4500 l of milk at 3.6% butterfat and 8.6% solids-not-fat’ (ADAS, 1983; DEFRA, 2010). Adjusting the number of LSU in each system based on corrections for live weight and productivity stipulated by ADAS (1983), differences in observed cow performance amongst systems were embedded in the FU. From rank analysis, the emissions intensity of LFC was lower than that of the higher yielding LFS system. However, S genetic line animals had a higher feed and metabolisable energy intake and greater milk yield than those of the C line. This led to the S line having higher enteric methane emissions per cow and higher gross emissions associated with both forage and purchased feeds. Unlike using ECM as the FU for emissions intensity, the higher gross emissions associated with the S line were not sufficiently offset by the higher productivity when using LSU.

Milk yield corrected for milk fat and protein content is the most commonly applied FU in LCA studies of dairy production. Employing ECM incorporated the effect of disparity in milk production amongst systems when examining the GWP. This was also the only FU to find the emissions intensity of all four Langhill systems to be significantly different from each other. In a recent study in Ireland, O’Brien *et al*. (2012) found that a confinement system had higher emissions intensity than a grazing system per kilograms ECM. Our results disagree with these findings, but there were several differences in farm-management practices and methods between the studies. The Irish systems had substantially lower milk yield than the Langhill systems, and a further key difference was that 70% to 80% of concentrated feed components of the Irish diets were internationally imported. The latter included maize from the United States and rapeseed meal and molasses from Germany, whereas in the Langhill systems, forage crops were grown on-farm, and the purchased components of the TMR were largely sourced from the same country. It is important to recognise that results of studies, in particular those of HF and LF systems in our study, depend on site-specific conditions, such as the purchase of certain concentrated feeds or the ability to grow certain crops locally. Langhill rations sourced a high percentage of their concentrated feeds from by-product grain from distilling and brewing industries, which has a considerably lower GHG emissions intensity than palm oil or soyabean meal imported from South America (Carbon Trust, 2010). Countries or regions differ considerably in their management preferences, climatic conditions, soil types, and availability or feasibility of crops. Brockman and Wilkins (2003) described variation amongst grass species in growth patterns, nutritional content, response to nitrogen and climate, and most importantly, yields. In turn, these factors influence the range of animal breeds and management systems available to dairy farmers, as well as the environmental impacts associated with them. This point highlights the importance of examining site-specific methods and farm-management practices when comparing LCA studies which use the same FU.

Milk solids are another unit commonly used in the dairy industry, for example to compare the biological or production efficiencies of cows, but seldom used in LCA. This is likely because most dairy LCA studies draw their boundaries at the farm gate, at which the principal product is liquid milk. Exceptions to this have occurred in studies from countries where output of alternative dairy products such as dried milk, whey powder and butter exceeds that of liquid milk, such as New Zealand and Ireland. This again is pertinent to the examination of site-specific practices. For studies interested in the life cycle of dairy-derived products such as yoghurt or cheese, referencing the GWP to MS may also be more appropriate. When using units which included dairy production (ECM and MS), results followed a similar trend, with LFS having the lowest emissions intensity and HFC the highest. However, unlike using ECM, there was no difference observed in GWP between LFC and HFS when using MS. This was likely due to a confounding effect introduced by the interaction of forage regime and genetic line in the production systems. Both fat and protein contents of milk were higher in HF systems, while milk yields were higher in the LF systems. This was not unexpected, as cows on a LF regime have historically been subject to milk-fat depression (Bauman and Griinari, 2001). Both milk yield and MS were higher with the S genetic line, consistent with its genetic potential. Thus while the LFC system yielded more raw milk containing fewer MS, the HFS produced less milk containing more MS. Although calculating ECM corrected for differences amongst systems’ milk fat and protein contents, the impact of this confounding effect upon GWP was more pronounced when MS was the FU. This emphasises the importance of considering not only the GWP results, but also what information the FU may contain or tell about the dairy production systems examined.

Using land use as the FU satisfies the conventional definition of efficiency as a measure of system output to system input. Further, land area conforms to the ISO 14040 stipulation that the FU be a clearly defined and measurable reference to which input and output data are normalised (ISO, 2006). For studies which intend to inform national GHG inventory reporting, it is also necessary to choose an FU coupled with land for area-based processes (IPCC, 2006). In agriculture, this may include emissions associated with applied fertilisers and crop production and residues, but it is not a requirement for animal emissions such as enteric CH_4_. The LCA process may include within its boundary emissions sources beyond those required for national inventory reporting; thus, an area-based unit is not a specific requirement of LCA. Life-cycle assessment studies have variously referenced GWP to on-farm pasture, combined on-farm pasture and forage-crop land, or to total on- and off-farm land, including that required to produce imported concentrated feeds and bedding (Haas *et al*., 2001; van der Werf *et al*., 2009; O’Brien *et al*., 2012). In our study, on-farm land was the only FU for which both HF systems were more efficient than both LF systems. High overall GWP combined with lower on-farm land use due to the absence of grazing meant that LF systems had higher GWP per hectare of on-farm land. In HF systems, higher on-farm land use resulted in lower GWP per hectare. From an LCA point of view, including only on-farm area is not appropriate for examining results as arable land used to produce purchased feed, which could be located worldwide, will also be responsible for environmental impacts in the life cycle of milk production (Yan *et al*., 2011). Thus, the GWP reported for conventional dairy production systems are often inexorably linked to the emissions intensities of crop production abroad.

As noted earlier, an FU serves as a measurable reference to which input and output data are normalised. In the same way that milk yields can be adjusted according to their fat and protein contents to ensure functional equivalence, should productive areas of land be adjusted in LCA to normalise their productivities across different locations? In doing this, GWP per hectare would no longer reflect differences in crop yields, but only differences in downstream system specific factors such as animal genetics, the formulation of rations, and other farm-management factors. Noting very different areas of land used to produce equivalent quantities of grain, Audsley *et al*. (1997) equated land areas of different systems by assuming that the difference in land used was managed as set-aside land (Audsley *et al*., 1997). Wackernagel and Rees (1996) introduced the notion of a ‘global hectare’ with their concept of the Ecological Footprint. To equate land around the world, they quantified demand on biological resources by expressing all components of an impact as an equivalent area of land and sea with world average productivity. Yield and equivalence factors were used to convert actual physical areas from local hectare to global hectare. Scaled down to the level of agricultural systems, yield factors, obtained by dividing the local yield of a biological product by its global average yield (Wiedmann and Lenzen, 2007), can account for differences in productivity of a given crop and land type amongst countries.

Increasing efficiency of livestock production by improving genetic potential has been identified as a promising approach for reducing global emissions from livestock systems (Steinfeld *et al.*, 2006). In the Langhill systems, S animals were bred to be in the top 5% of UK genetic potential for milk fat and protein contents. Previous studies have noted lower emissions intensity of S systems for both enteric methane (Chagunda *et al*., 2009) and overall GWP per unit ECM (Ross *et al*., 2014). When using an area-based FU, however, results did not differ between C and S genetic lines managed under the same forage regime. Selected animals consume more feed than C animals and consequently require more land and crops to produce their feed. Thus, as milk production, emissions and land use increase, using an area-based FU does not reflect the specific improvements made by the Langhill selection criteria. This is not so much a problem of the FU as it is reflection of what the FU reveals about the systems. The total land FU is a valid measure of agricultural or emissions intensity, and, in the event of no significant difference between systems under study, one could look to production or other criteria to evaluate systems.

Some LCA studies of dairy production have used an FU unique to the study, such as ‘1000 euros of gross farm income’ (van der Werf *et al*., 2009). As the selection of an FU often remains at the discretion of researchers, they have the opportunity to innovate new and diverse FU to provide a balanced or new perspective. These FU could incorporate a socio-economic aspect such as the monetary unit employed by van der Werf *et al*. (2009). The use of either ECM or MS as the FU, rather than raw milk yields, enables a study to account not just for milk quantity but quality, which in turn can translate into milk price. Considering the observed differences in emissions intensity between HF and LF systems, a farm-management decision to implement a given system, made for economic reasons, inexorably influences the system’s environmental impacts. Feed costs can account for around 80% of total variable costs of milk production (Shalloo *et al*., 2004) and around 35% of the environmental impacts of the Langhill systems in this study (Ross *et al*., 2014). In future, LCA should perhaps consider an FU which can reflect economic functions of a system, such as ‘GWP per 1000 euros of gross farm income’ (van der Werf *et al*., 2009) in addition to the production or land use. Alternatively, an FU could reflect competition for resources (e.g. per kilogram of non-human-edible feed) or reflect efficiency (e.g. per MJ of potential energy of system inputs such as fuel, feed). However, the perceived performance of any production system as a function of efficiency may have to do with the different ways in which efficiency is understood. For example, efficiency in an animal production system is commonly defined by its feed conversion ratio. This also raises the question of whether FU selection should be tailored to the intention of a study or use a widely employed, standard FU. Further, an FU in LCA should be relevant to reference a wide range of impact categories. Haas *et al*. (2001) stated that environmental impacts at a regional or local level, such as nitrate or phosphate pollution of a lake, have a strong area-related aspect and must be minimised irrespective of production levels that farmers can achieve. Given the multi-functionality of agricultural systems, LCA results should be evaluated using both production and land-based FU to provide a balanced assessment.

The new FU developed in our study aimed to account for both the productivity and the total on- and off-farm land use of a system. Although LFS had significantly lower GWP per kilogram ECM, when using ECM/Land_total_ as the FU, there was no significant difference between the emissions intensity of LFC and LFS. Indeed, much like the two FU based on land use, there was no significant difference between systems under the same forage regime. Given the range of milk yields in the Langhill systems, it was perhaps surprising that using the dual FU was able to differentiate only between the emissions intensities of feeding regimes but not those of genetic lines. Despite incorporating milk yield, the using this FU did not reflect the difference amongst systems’ GWP that the existing FU incorporating productivity were able to determine. However, when analysing the systems’ relative efficiency rankings in our study, the dual FU did find LFS to have significantly lower GWP than LFC.

It has been noted in the literature that there is a lack of significant correlation between GWP per unit product and GWP per unit land (Casey and Holden, 2005). Yan *et al*. (2011) defined how this correlation could be achieved with an equation incorporating the milk yield per cow, the stocking rate and ratio of on-farm to off-farm land use. We propose that using the dual FU can measure the outcome of trade-offs, for example an improvement in emissions intensity using ECM as the FU being accompanied by a deterioration when using Land_total_, when assessing conversion from one production system to another. In the present study, a decrease in GWP per the dual FU would be observed in either a win–win scenario, in which both measures improved, or a trade-off with positive outcome, in which GWP per the dual FU would decrease despite an increase in GWP per hectare, for example. Thus the remaining scenarios, trade-off with negative outcome and lose–lose, would be reflected by an increase in GWP per the dual FU. In the context of the present study, a conversion from HFC or HFS to either LFC or LFS represent win–win scenarios, with a decrease in GWP per ECM, per Land_total_ and the dual FU. A conversion from HFC to HFS represents a positive outcome, with a decrease in GWP per dual FU despite an increase in GWP per Land_total_. Godfray *et al*. (2010) stated that there is a pressing need in modern global agriculture for ‘sustainable intensification’, in which yields are increased without adverse environmental impact and without cultivating more land. To comply with this definition, it would be advantageous for LCA of dairy production systems to use an FU which could account for environmental impacts relative to yield and land use simultaneously. In our study, the dual FU yielded results broadly consistent with those using ECM or total land use as the FU, and helps to assess the likelihood of a positive trade-off between GHG emissions and efficiencies of production and land use. After investigating a similar concept of trade-offs between GWP per hectare and ECM per hectare, the study of Hayashi (2013) recommended that both expressions should be used complementarily. It is advisable that, for an appropriately balanced assessment, LCA should consider the underlying reasons behind trade-offs, and thus also evaluate emissions intensity per both land-oriented and product-oriented FU individually.

## Conclusions

The relative emissions intensity of different dairy production systems sometimes changed based upon the FU employed. Energy-corrected milk was the FU most effective for reflecting differences between the systems. Functional units that incorporated a land-related aspect found no difference between systems which were managed under the same forage regime, despite their comprising different genetic lines with considerably different productivity.

Results from LCA studies comparing dairy production systems should be considered in the context of the FU used, in order to appraise them in an informed manner. Energy-corrected milk yields should remain the primary FU for comparing impacts of dairy production systems, however, both a land-use-based FU and the dual FU should be used in addition, in order to evaluate trade-offs and present a balanced assessment.
